# A Novel 3D Printed Titanium Implant for Anterior Cervical Discectomy and Fusion

**DOI:** 10.7759/cureus.3952

**Published:** 2019-01-24

**Authors:** Levonti Ohanisian, Michael J Dorsi

**Affiliations:** 1 Miscellaneous, Florida Atlantic University, Boca Raton, USA; 2 Neurosurgery, Thousand Oaks Spine Institute, Thousand Oaks, USA

**Keywords:** acdf, interbody, spondylosis, 3d printed titanium implant

## Abstract

Cervical spondylosis is a common age-related disorder that results in pain, radiculopathy, and myelopathy. A retrospective chart and radiograph review of a 50-year-old male who underwent surgical treatment for correction of cervical radiculopathy was performed. Immediately after surgery the patient reported complete relief of his preoperative arm pain. In two weeks, he had recovered full strength and sensation. Six months postoperatively, the patient reported relief of all pain and complete recovery of strength and sensation. Anterior cervical discectomy and fusion (ACDF) with an open architecture titanium implant was successfully utilized to improve cervical radiculopathy. This technique increases the likelihood of fusion and improved patient outcome. The objective of the report is to highlight the treatment of cervical radiculopathy through anterior cervical discectomy and fusion with a 3D printed titanium alloy with an arched design and large porous openings. This is one of the first reports using this interbody device in a multilevel procedure.

## Introduction

The most common cause of neural dysfunction in the cervical spine is attributed to spondylosis. This age-related change can result in symptoms directly through disc herniation, osteophyte formation, and a hypertrophied ligament compressing the neuraxis [1].^ ^The resulting symptoms include pain, radiculopathy, and myelopathy. First line therapy is conservative; however, patients are surgical candidates once they have failed conservative therapy. The mainstay surgical treatment for cervical radiculopathy is anterior cervical discectomy and fusion (ACDF), which is currently the most commonly performed surgical procedure for degenerative cervical spine disease [2]. ACDF is a very well tolerated operation. A retrospective study by Fountas et al. involving 1051 patients reported a mortality rate of 0.1% and noted the most common complication of the operation to be isolated postoperative dysphagia [3].

The implants used in ACDF have undergone dramatic changes since Cloward, Robinson, and Smith introduced the anterior approach using a dowel graft in the 1950s. Cage fusion technology was first introduced in 1988 by Bagby and it too has undergone rapid changes [4]. Current cages enhance osteogenesis and thereby fusion through using titanium to increase surface roughness. Titanium is modified as such through plasma beam and electron spray techniques [5].^ ^Increases in osteogenic cell differentiation have been demonstrated through this modification by increasing total protein and alkaline phosphatase levels [6]. Cage technology has proven to reduce morbidity and be very effective in ACDF [7]. This paper presents a case report of ACDF using a titanium open architecture cervical interbody and the relative surgical advantage of using this type of device. This is one of the first operations in which such an interbody was used in a multilevel procedure.

## Case presentation

A 50-year-old male presented with a two-month history of progressive right arm weakness and pain. The initial presenting symptom was a stiff neck with intermittent low-grade neck pain that developed into severe right shoulder pain. Several weeks after the onset of pain, he noticed diffuse right arm weakness and loss of muscle tone that persisted. Upon presentation, he reported painful paraesthesia in C6 dermatomal distribution. Motor examination found diffuse weakness in the right arm: shoulder abduction 4/5, elbow flexion 4/5, elbow extension 5/5, finger flexion 4/5. He was hyperreflexive in all extremities.

**Figure 1:**
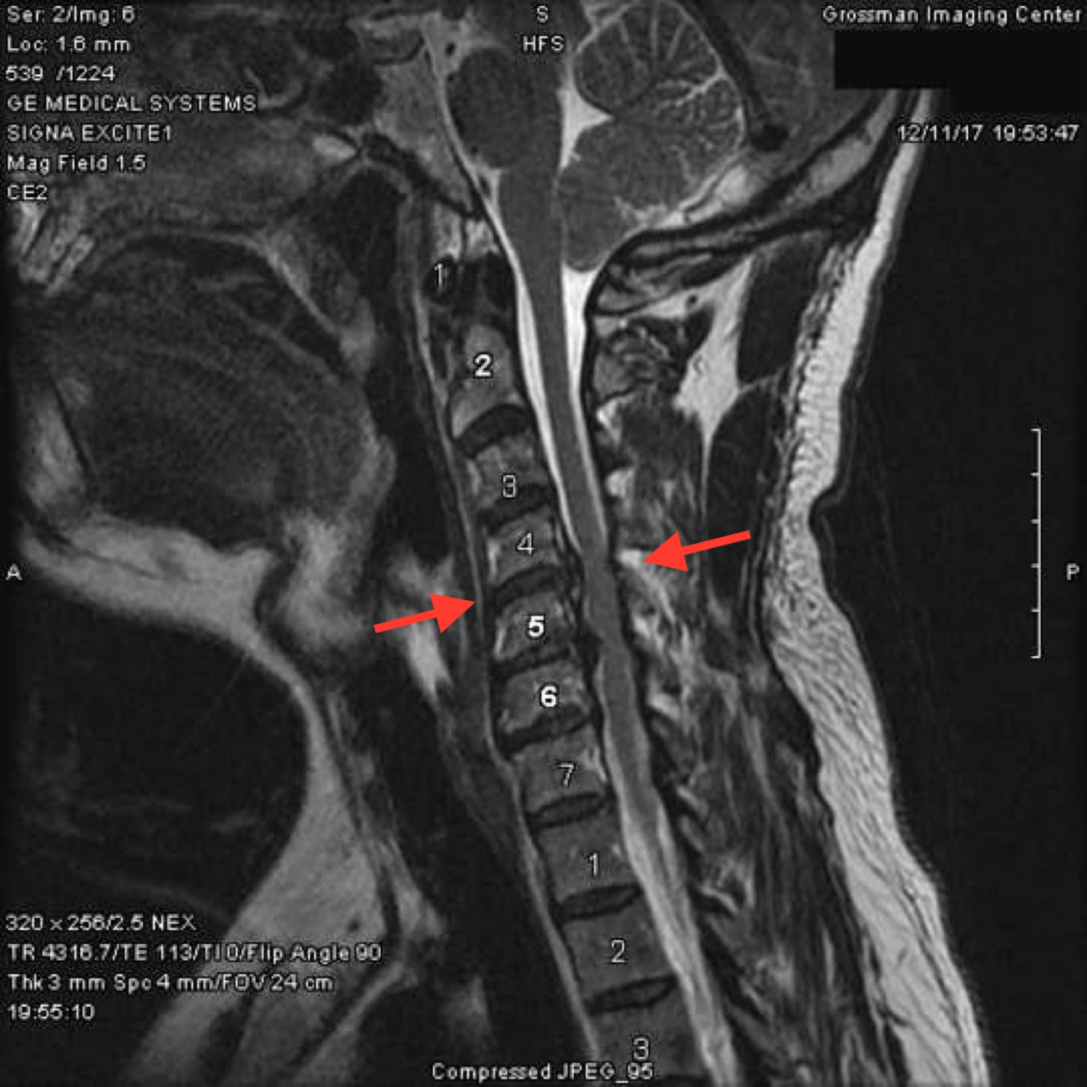
Multilevel degeneration and stenosis most pronounced at C4-5

**Figure 2:**
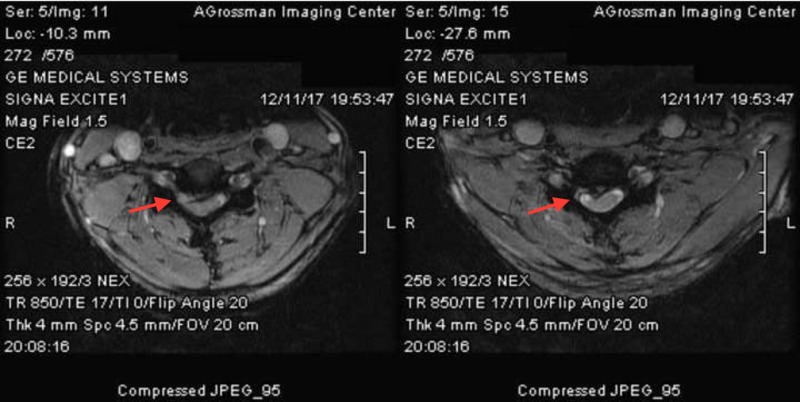
Axial MRI of cervical spine Retrolisthesis with 5 mm right paracentral disc herniation extending into the foramen (left) and C5-6. A right paracentral disc extrusion compressing the right anterior aspect of the spinal cord (right).

Magnetic resonance imaging (MRI) of the cervical spine (Figure 1) showed multilevel degeneration and stenosis most pronounced at C4-5: retrolisthesis with 5 mm right paracentral disc herniation extending into the foramen (Figure 2) and C5-6: a right paracentral disc extrusion compressing the right anterior aspect of the spinal cord (Figure 2). Electromyography/nerve conduction studies (EMG/NCS) demonstrated subacute right C5 and C6 radiculopathy along with chronic right C7 radiculopathy.

C4-5 and C5-6 anterior cervical discectomy and fusion with placement of a titanium interbody (Spira-C, Camber Spine, Wayne, NJ) and titanium plate and screws (Depuy Spine, Raynum, MA) was performed.

Postoperative course

**Figure 3:**
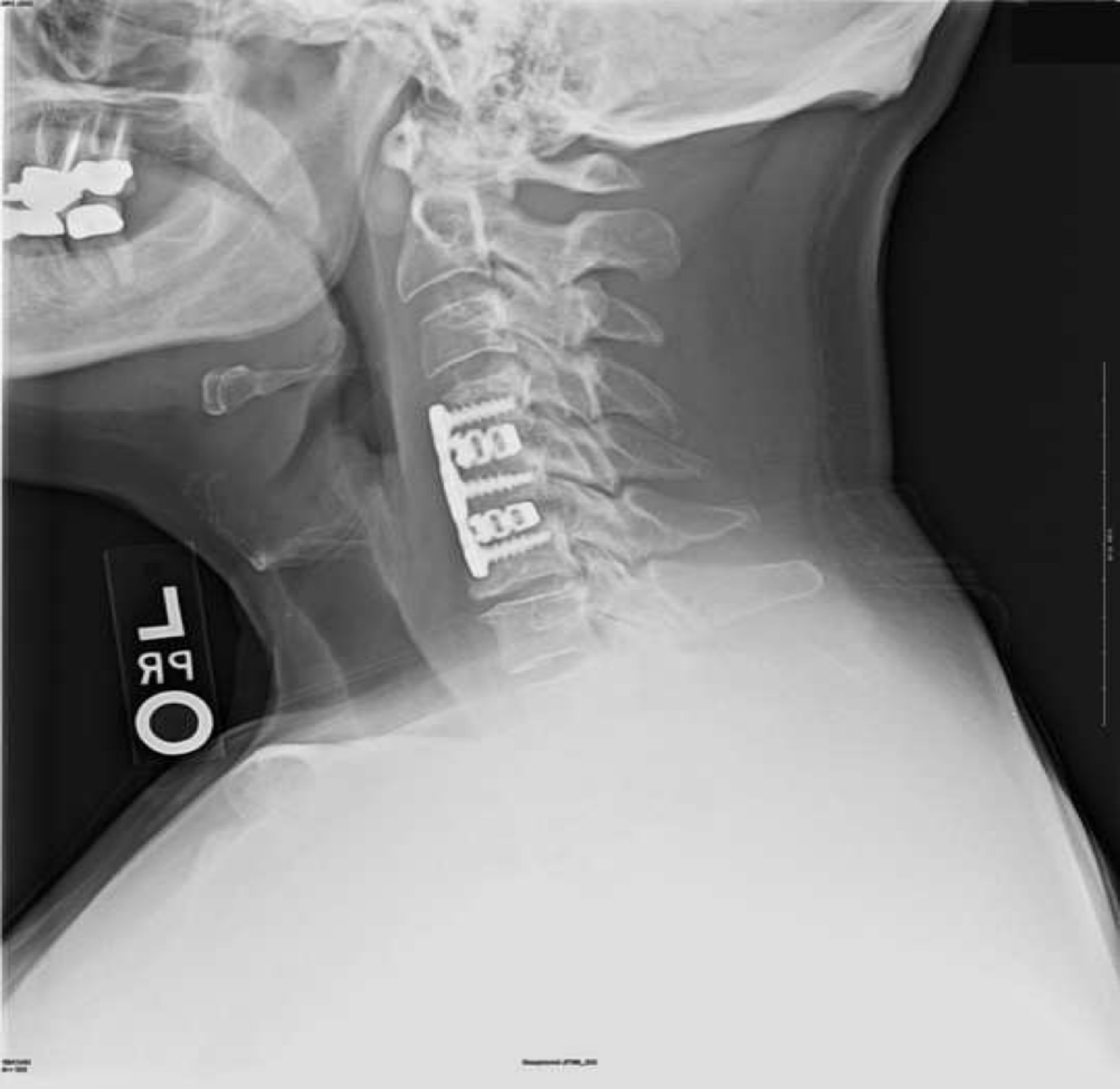
Lateral X-ray at four months showed improved alignment, resection of posterior osteophytes, and early graft incorporation

**Figure 4:**
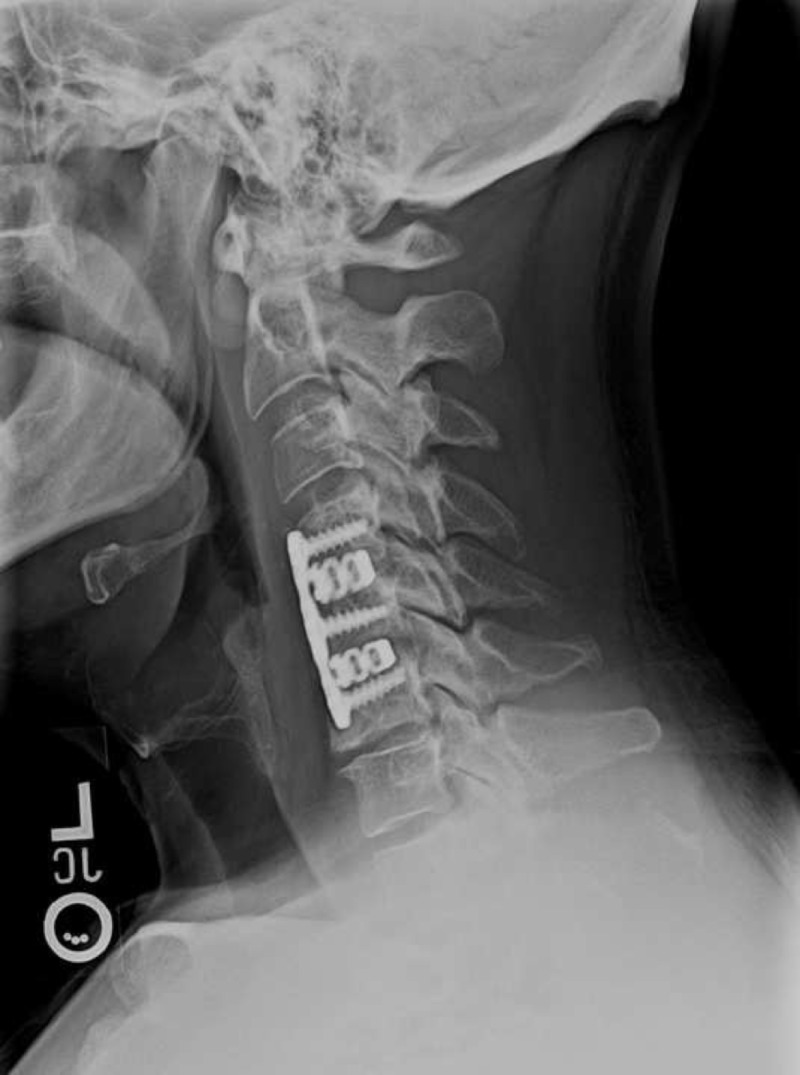
Lateral X-ray of cervical spine at nine months

Immediately postoperatively the patient had relief of arm pain and improvements in strength and sensation. This was maintained for nine months after surgery. Two weeks postoperatively, the patient’s symptoms and neurologic function improved drastically. Greater right arm strength was noted with 5/5 strength in upper and lower extremities bilaterally. Right shoulder abduction was 5/5 and external rotation 4+. Six months postoperatively, the patient reported relief of all pain and complete recovery of strength and sensation. At the nine month follow-up, the patient remained completely asymptomatic. A lateral X-ray at four months (Figure 3) showed improved alignment, resection of posterior osteophytes, and early graft incorporation. A lateral X-ray at nine months (Figure 4) continued to demonstrate similar findings.

## Discussion

The goals of anterior cervical discectomy and fusion are to achieve neural decompression, maintain cervical lordosis, provide segmental stabilization, and eliminate symptoms of radiculopathy. Interbody spacers are created using a variety of materials and in different designs [8-11]. Furthermore, osteogenic substances may be added to enhance intervertebral bone matrix formation [10,12,13]. Osteogenesis and thereby fusion can be enhanced through the use of titanium to increase surface roughness. Titanium surfaces may be modified through plasma beam and electron spray techniques [5]. Such modifications increase total protein and alkaline phosphatase levels, which enhance osteogenic cell differentiation [6].

This was one of the first operations in which an open matrix cervical interbody device was used in a multilevel procedure. There are advantages of using a roughened titanium surface as opposed to others such as smooth titanium alloy or poly-ether-ether-ketone (PEEK). PEEK does not integrate well with the surrounding bone and instead may form a fibrous connective interface [14-17]. A 2013 paper by Olivares-Navarette et al. demonstrated that roughened titanium is helpful for bone formation and remodeling. This is done through stimulation of osteoprotegerin, TGF-β1, VEGF-A, FGF-2, and angiopoietin-1 production. Levels of these factors were greater on rough titanium alloy than both smooth titanium alloy or on PEEK [14], [18].

## Conclusions

Interbody devices have undergone enormous evolution since their inception in the 1950s and have improved clinical outcomes of anterior cervical discectomy and fusion (ACDF). ACDF is currently the most common surgical intervention for degenerative cervical disease. It is a very well-tolerated operation that continues to see improvement through advancements in research and technology. Our case report illustrates ACDF in a patient, using a porous titanium interbody device that stimulates osteogenesis and enhances fusion, with a remarkable improvement of symptoms.
